# LZS-1, Lanzarote (Canary Island, Spain) lunar (Apollo 14) basaltic soil simulant

**DOI:** 10.1038/s41598-022-20960-8

**Published:** 2022-10-01

**Authors:** Fernando Alberquilla, Jesús Martínez-Frías, Valentín García-Baonza, Rosario Lunar

**Affiliations:** 1 Instituto de Geociencias (CSIC, UCM), Calle del Dr. Severo Ochoa, 7, 28040 Madrid, Spain; 2 Departamento de Química Física, Facultad de Ciencias Químicas, Universidad Complutense de Madrid, 28040 Madrid, Spain; 3 Instituto Volcanológico de Canarias (INVOLCAN), San Cristóbal de La Laguna, Tenerife, Canary Islands Spain

**Keywords:** Mineralogy, Petrology, Ceramics, Mechanical properties

## Abstract

The search for Terrestrial Analogues is essential for the development of future permanent or semi-permanent lunar bases. Terrestrial Analogues are zones where it is possible to probe not only scientific instruments, but also other astronaut capabilities in an environment that is similar to the geological context, geomorphology, mineralogy, geochemistry, etc. that we can find on Mars, the Moon and even asteroids. This work has focused on a multi-analytical characterization of Peñas de Tao geosite basalts in Lanzarote (Canary Islands, Spain). This characterization starts from a field campaign in which 3000 g of basalt rocks were selected. Subsequently, they were analysed by different techniques to determine their composition at a mineralogical and geochemical level, and the results were compared with data from other lunar simulants and from the Apollo 14 mission. After that, a set of petrophysical tests was carried out in order to determine its physical properties and evaluate its capacity as an analogous material for use in situ as a resource for further geological and astrobiological (future lunar habitability) essays.

## Introduction

A lunar soil simulant can be considered as a type of geological material that is similar to lunar regolith for its mineralogical, chemical, and mechanical properties^1^. Currently the supply of materials for space missions is very expensive and limits their development^2^. For this reason, the establishment of a future semi-permanent lunar base will require the use in situ of regolithic material as a resource^3^. This regolith covers a wide spectrum of uses; from its use for the construction of habitability modules, roads, landing areas … etc., and as a source of supply to obtain other essential resources for the survival of astronauts such as oxygen or water^2^.

Since in 1990 a group of scientists from the United States developed the first standardized lunar regolithic simulant called JSC-1 (Johnson Space Center), others with different functions and goals have been developed^4^. According to Zheng et al. (2009)^5^ some of the regolithic simulants mentioned are MLS-1 (Minnesota Lunar Soil, developed by Weiblen & Gordon, 1998)^6^, FJS-1 and MKS-1 (developed by JAXA; Japan Aerospace Exploration Agency) and CAS-1 (developed by the China Academy of Sciences). However, most of these simulants are either already exhausted (JSC-1, MLS-1), or can only be used from an engineering and non-scientific point of view (FJS-1 and MKS-1)^5^. In this sense, a new lunar simulant, LZS-1, has been developed in Spain. Its mineralogical and geochemical characteristics and petrophysical properties are described in this paper.

Tao geosite^7^ in Lanzarote (Canary Islands, Spain) was selected for this work. Lanzarote is made up of a basement formed in a continental slope environment and an approximate age of between 66–55 m.a. It was not until 33 m.a ago (Oligocene) when the underwater volcanism began, and 15.5 m.a ago the emergence of the island took place^8^.

**Figure 1:**
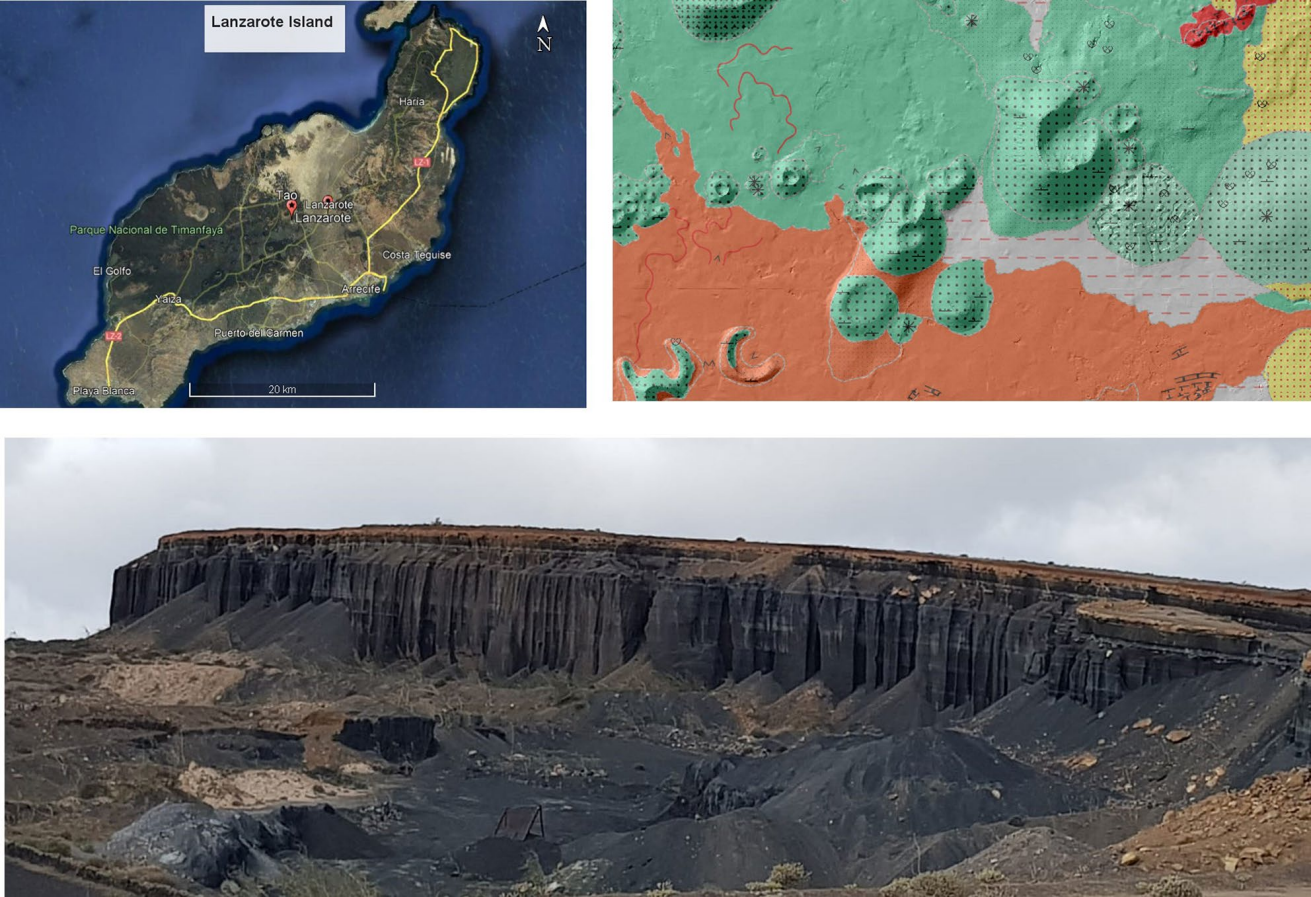
(**A**) Geographic map of Lanzarote (Canary Island) and position of the Tao sampling area. (**B**) Geological map of Tao. Green colours represent basaltic tephra cones and scattering pyroclasts. The red colours represent basalt and basalt olivine flows (Canary Islands Government. IDECanarias Visor 4.5.1. Available online: https://visor.grafcan.es/visorweb/#). The yellow colours are sandy deposits and the grey sandy and clayey alluvial deposits. C) Tao quarry.

According to Romero et al. (2019)^9^, and from a chronostratigraphic point of view, the island of Lanzarote has been divided into four volcanic series (Series I, Series II, Series III and Series IV). Peñas de Tao together with Timanfaya which is one of the biggest lava flows in the world^10^, belongs to the set of historical volcanic eruptions that took place on the island during the seventeenth and nineteenth centuries. Peñas de Tao (Fig. 1) is located right in the middle of the island, in the vicinity of Tamia Mountain (a horseshoe-shaped eruptive volcanic complex of middle Pleistocene age). In general, the magmatic evolution of the island ranges goes from strongly alkaline and ultrabasic terms in the first series (I, II, III) to alkaline-subalkaline and basic terms^11^ in the set of historical eruptions (series IV).

## Background and lunar analogues

We know that there are three different types of soils on the lunar surface. There are lunar mare, highlands, and mixing zones^12^. In this work, we identify which region of Lanzarote Island shares the greatest similarities with some of the lunar soil types^13,18^, according to the contributions of samples from NASA's Apollo missions. After that, we developed one of the two recommended types of lunar regolithic simulants, low-Ti mare basalt and high Ca highland anorthosites^5^. In this case, we manufacture the low titanium regolithic simulant. The main goals are the in-situ use of lunar regolithic simulant as a resource in civil engineering works (roads, paths, habitation modules, runways … etc.) and due to the low percentage of heavy metals, we can prove it as substrate for growing food^14^. As secondary goals: the regolithic simulant can be used to test the capacities of the human being to extract its direct resources^15^ such as: oxygen (to provide life support astronauts), iron, titanium, or chromium (structural elements) and helium (fuel).

**Figure 2:**
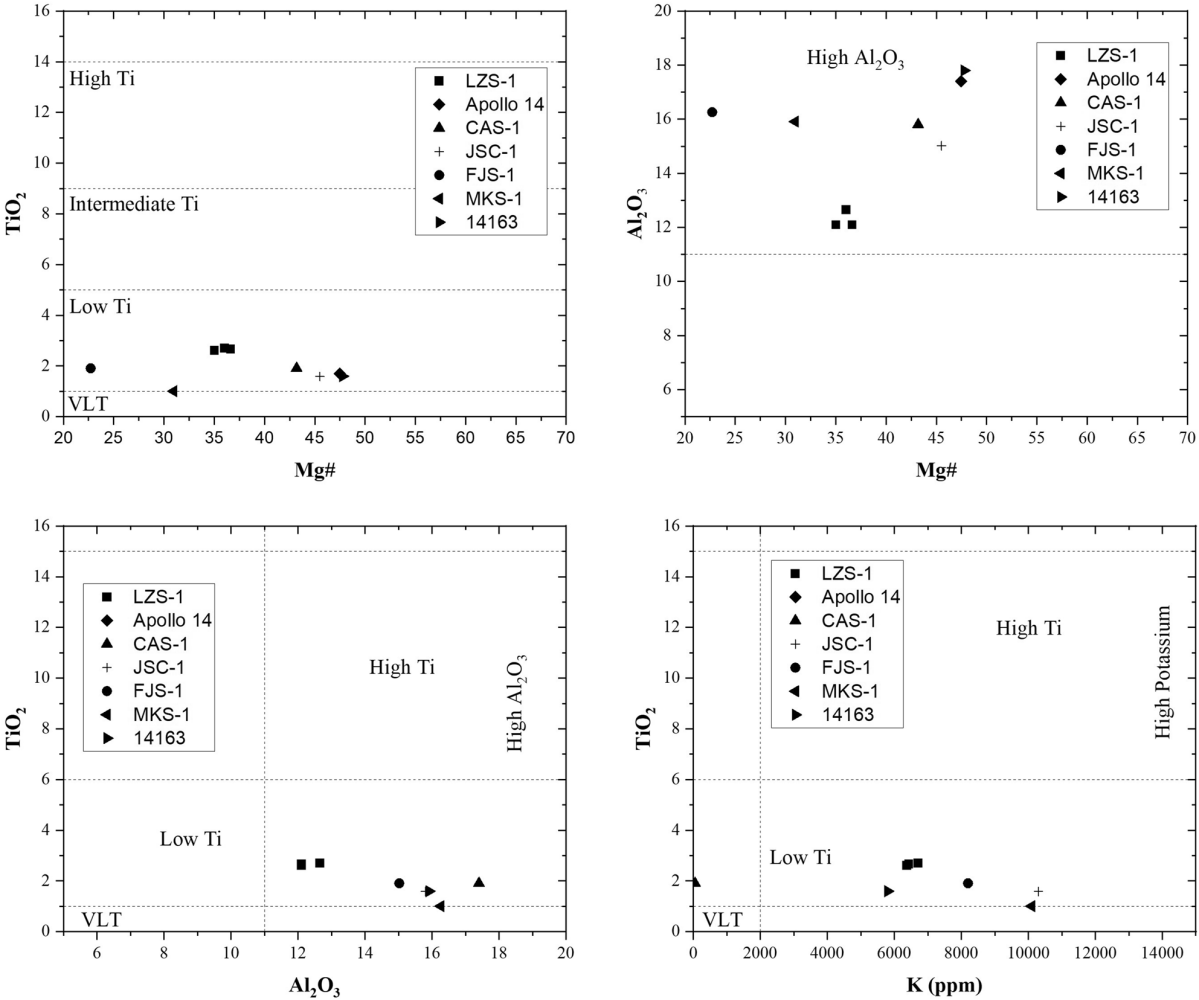
Classification diagrams of the lunar regolithic simulants and samples of LZS-1, Apollo 14, CAS-1, JSC-1, FJS-1, MKS-1 and 14,163 (Zheng et al.^5^) according to their content in Mg # (Mg / Mg + Fe) TiO_2_, Al_2_O_3_ and K (ppm).

**Figure 3:**
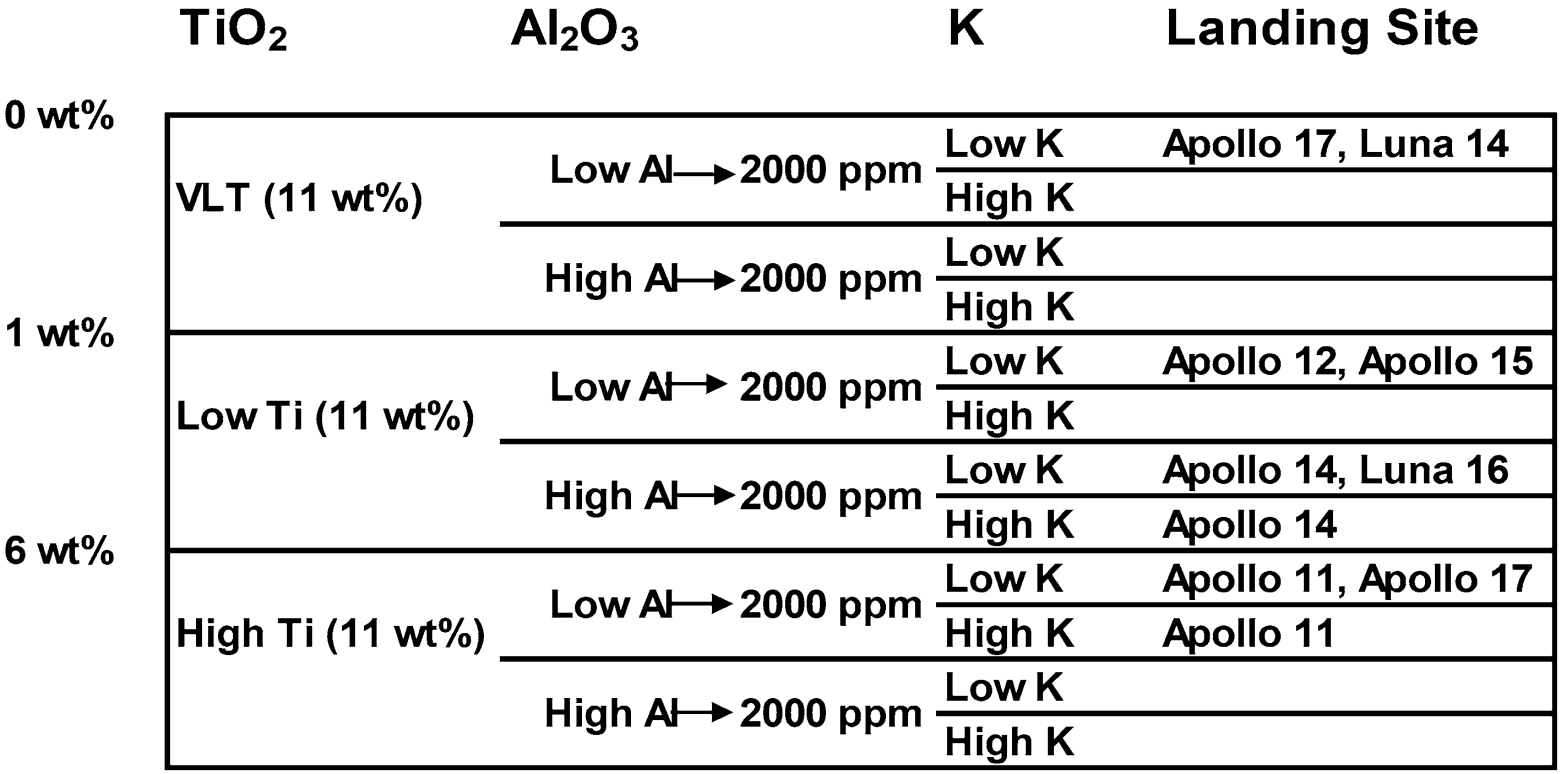
Neal & Taylor^16^ classification diagram, where it is compared the values of Fig. 2. to establish a direct relation between the different regolithic simulants and the lunar landing site.

To establish the relationship between the study area and the lunar surface we resort to the classification of Neal & Taylor (1992)^16^ where we can deduce to which landing site our samples correspond according to their content of Titanium, Aluminium and Potassium (Fig. 2). According to Fig. 3 we can establish a direct analogue with the samples from the Apollo 14 mission (Fig. 3). This lunar landing site is known as the Fra-Mauro region and corresponds to a mixing zone located on the border between a mare soil and a highland soil^5^.

## Lanzarote basaltic simulant LZS-1

### Material and methods

In the field campaign, 3000 g of basaltic rocks were collected from Tao quarry. From them, to carry out the analyses, 1500 g “fresh” basalt samples that did not show alteration were selected, trying to represent their different textural characteristics (e.g. massive, vesicular). For the sampling, we had the appropriate permits for access to certain exploration areas, thanks to the support of the UNESCO World Geopark of Lanzarote and Chinijo Archipelago^17^.

**Figure 4:**
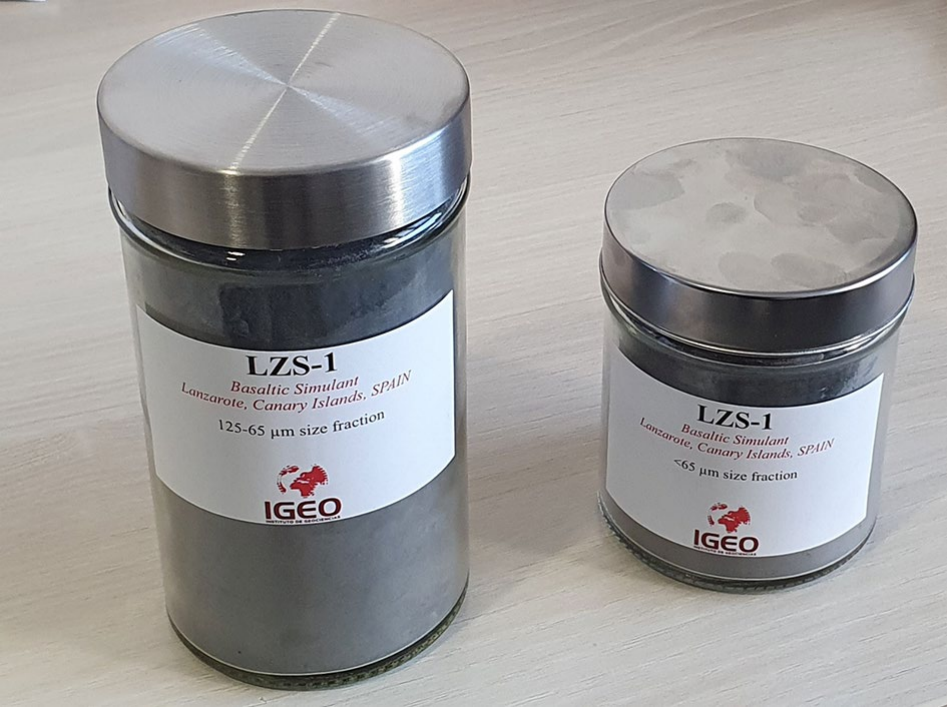
LZS-1 Lanzarote basaltic simulant.

**Figure 5:**
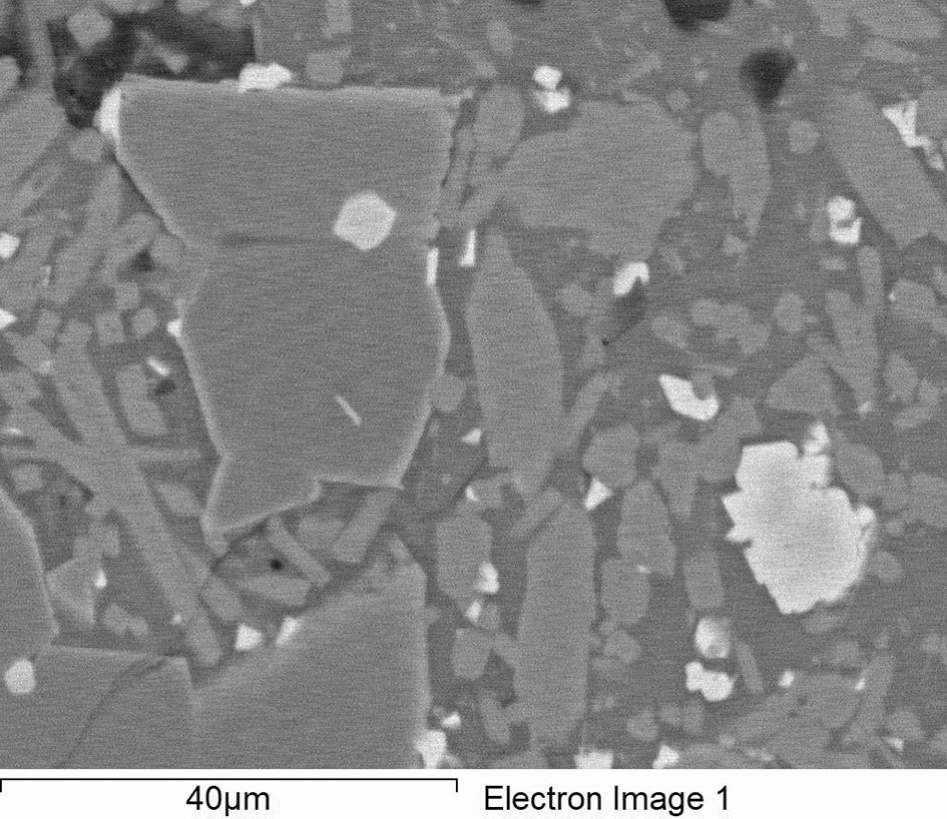
Scanning electron microscope image showing the size and shape of the LZS-1 grains.

In accordance with the results obtained in previous articles^18–21^, it was decided to sieve the 1500 g “fresh” basalt samples into two main fractions in order to represent the principal grain size distribution ranges of the lunar soil, in accordance with the most frequent ranges (Table 1). Figure 4 shows two cans of regolithic material. One belongs to the fraction between 63–125 µm (1000 g) and the other represents the fraction < 63 µm (500 g). With regards to the grain shapes, there are mainly angular (irregular) (Fig. 5), matching the results obtained by Katagiri et al. (2014)^22^ about grain shape characteristics of lunar soil.

**Table 1:** Mean and median values of particle sizes of lunar regolith at Apollo 11–17 landing sites and the regolith simulants CAS-1, JSC-1^5^, and LZS-1.

Sample	Particle size (µm)
Apollo 11 (median grain size)	48–105
Apollo 12 (median grain size)	42–94
Apollo 14 (median grain size)	75–802
Apollo 15 (median grain size)	51–108
Apollo 16 (median grain size)	101–268
Apollo 17 (median grain size)	42–166
CAS-1 (median grain size)	85.9
JSC-1	< 1000
LZS-1 (A)	63–125
LZS-1 (B)	< 63

For mineralogical and geochemical characterization, we perform the techniques of X-ray diffraction (Bruker D8 Advance diffractometer), X-ray Fluorescence (Bruker S2 Ranger spectrometer), Scanning Electron Microscopy Energy Dispersive X-ray (JEOL JSM-820), Electron Probe Microanalysis (JEOL Superprobe JXA-8900 M) and Mass Spectrometry with Inductively Coupled Plasma (Mass Spectrometer, with ICP ionization source, Bruker Aurora Elite). For the petrophysical characterization we carried out tests to determine the chromatic parameters (MINOLTA CM-700d/600d spectrophotometer). Determination of hardness according to the LEEB rebound method (EQUOTIP 3 Hardness Tester). Determination of surface roughness (TRACEIT optical surface roughness meter). Determination of the speed of ultrasound propagation (PUNDIT from CNS ELECTRONICS, Unit LPF-04-US). Determination of the porosity accessible to water, the real and apparent density, and the compactness. Determination of mercury-accessible porosity, pore size distribution, and tortuosity. Estimation of Uniaxial Compressive Strength (UCS). For the development of the LZS-1, basalt rock samples were first cut into regular shapes and placed in ovens for 48 h at temperatures of 60 °C until constant mass was reached. One third of the samples were reserved for petrophysical tests (superficial hardness, ultrasonic pulse velocity, mercury saturation and Hg porosimetry). The rest (2/3) was taken to a ball mill where 1500 g of regolith simulant were obtained. The sieving of basalt material has been standardized in the ASTM Standard in which the numbers 10 (2 mm), 18 (1 mm), 35 (0.5 mm), 60 (0.250 mm), 125 (0.125 mm) were used. To obtain fractions less than 65 µm, 500 g of the ground material was pulverized in an agate mortar and subsequently passed through a No. 230 (0.065 mm) sieve. Finally, it was stored in two different cans according to the fractions reached (Fig. 4).

### Petrography and mineralogy

Texturally, the samples are highly vesiculated, in which the percentage of vacuoles can reach 48% of the total in some cases. Its size is variable. The Tao samples have vesicle diameters from 150 µm to 2.5 mm. Other textures that we can differentiate are the vitreous (hypocristalline), aphanitic and microcrystalline textures.

**Figure 6:**
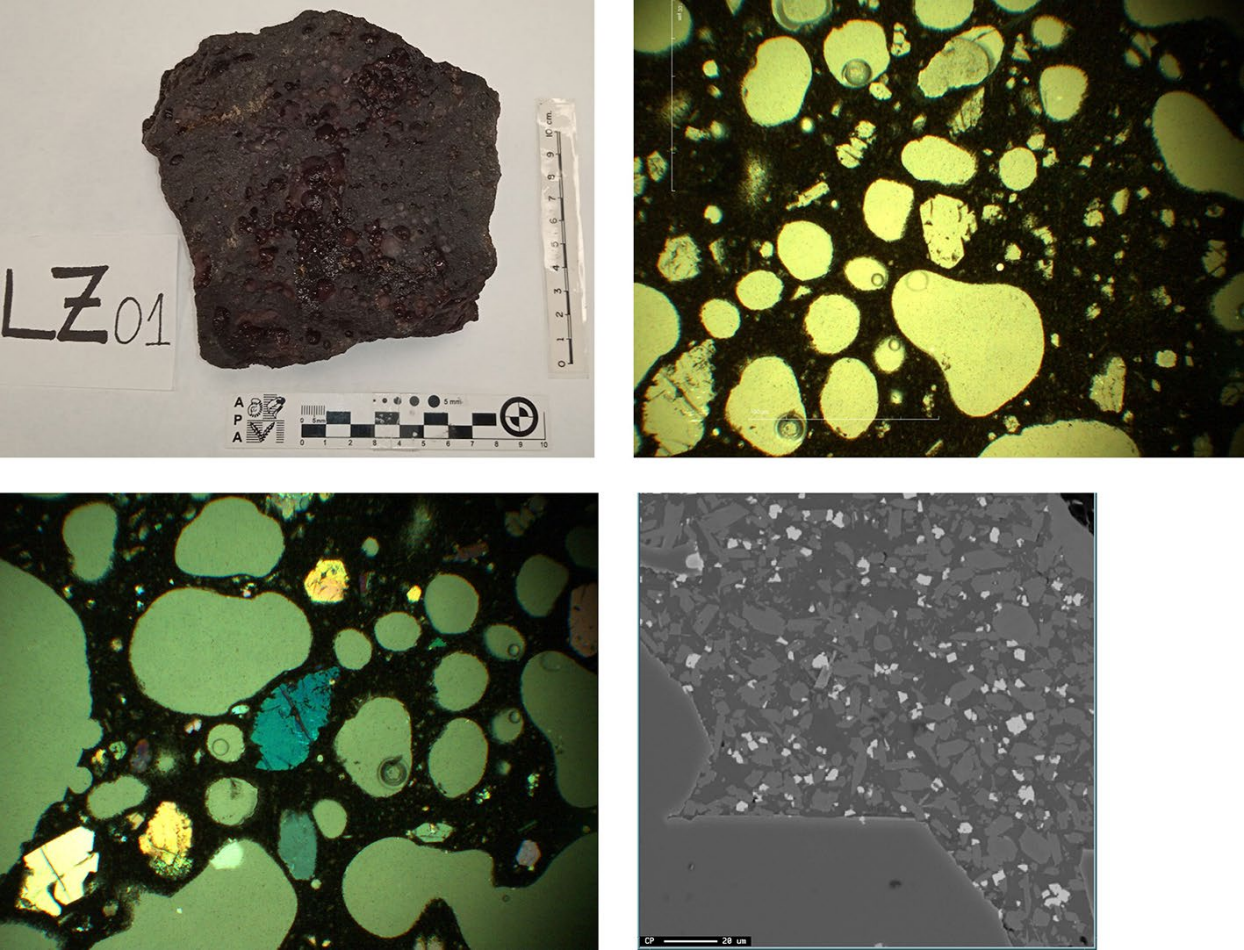
Image of the samples analysed on a macroscopic and microscopic scale with reflected light in parallel and crossed nickels and electron microprobe image. The blue and brown colours correspond to minerals from the olivine group. The black colour corresponds to the vitreous mass that has a mainly pyroxene composition. The data obtained from the electronic microprobe and the SEM–EDX has identified the main mineral phases.

Mineralogically there are three subclasses of silicates represented (Fig. 6); the nesosilicates: with olivine characterized by their marked relief, straight extinction, high birefringence, and rounded habit; the inosilicates with (clino-ortho) pyroxenes, tectosilicates with the plagioclase group (calcium) and glass principally constituted by pyroxene and plagioclase. On the other hand, there are metal oxides mainly of iron, titanium, and chromium.

**Figure 7:**
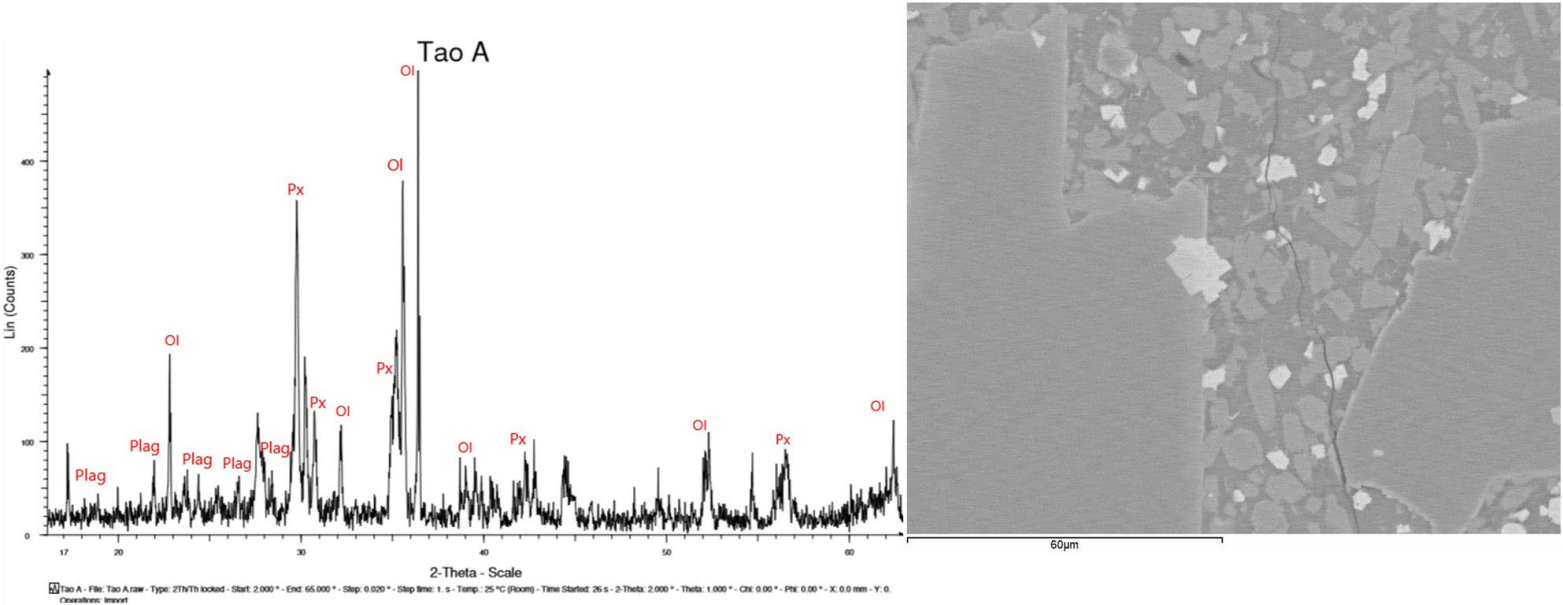
XRD data from LZS-1 and a detailed image of the SEM–EDX where the main mineral phases are represented.

Before carrying out any analysis, the samples were cleaned and dried in ovens at 70 °C until reaching a constant mass. After this, 10 g of basalt was crushed and ground for XRD (Fig. 7) and XRF. The rest of the chemical analyses were accomplished on the thin sheets carried out at the Research Support Center of the Complutense University of Madrid (CAI-UCM).

Major element composition is shown in Table 2, comparing it with that of CAS-1, JSC-1, FJS-1, and MKS-1. LZS-1 is similar to CAS-1, JSC-1 and to the Apollo 14 agglutinate sample 14163^5^.

**Table 2:** Major element composition of LZS-1 and compared with CAS-1, JSC-1, FJS-1, MKS-1 lunar soil simulant, and Apollo 14 lunar regolith^5^.

	SiO_2_	TiO_2_	Al_2_O_3_	MnO	MgO	CaO	Fe_2_O_3_	FeO	Na_2_O	K_2_O	P_2_O_5_	Total
LZS-1	45.6	2.7	12.3	0.2	8.2	10.6	14.6	–	2.9	0.7	2.0	99.6
Apollo 14*	48.1	1.7	17.4	0.1	9.4	10.7	–	10.4	0.7	0.6	0.5	99.6
CAS-1	49.2	1.9	15.8	0.1	8.7	7.3	–	11.5	3.1	1.0	0.3	98.9
JSC-1	47.7	1.6	15.0	0.2	9.0	10.4	–	10.8	2.7	0.8	0.7	98.9
FJS-1	49.1	1.9	16.2	0.2	3.8	9.1	–	13.1	2.8	1.0	0.4	97.7
MKS-1	52.7	1.0	15.9	0.2	5.4	9.4	–	12.3	1.9	0.6	0.1	99.5
14,163*	47.3	1.6	17.8	0.1	9.6	11.4	–	10.5	0.7	0.6	0.0	99.6

Apollo 14*, average chemical composition of lunar regolith sampled by astronauts at Apollo 14 landing sites. Data of Apollo 14* and 14163* were cited from Heiken et al. (1991)^23^, data of JSC-1 was cited from McKay et al. (1993, 1994)^18,24^.

**Figure 8:**
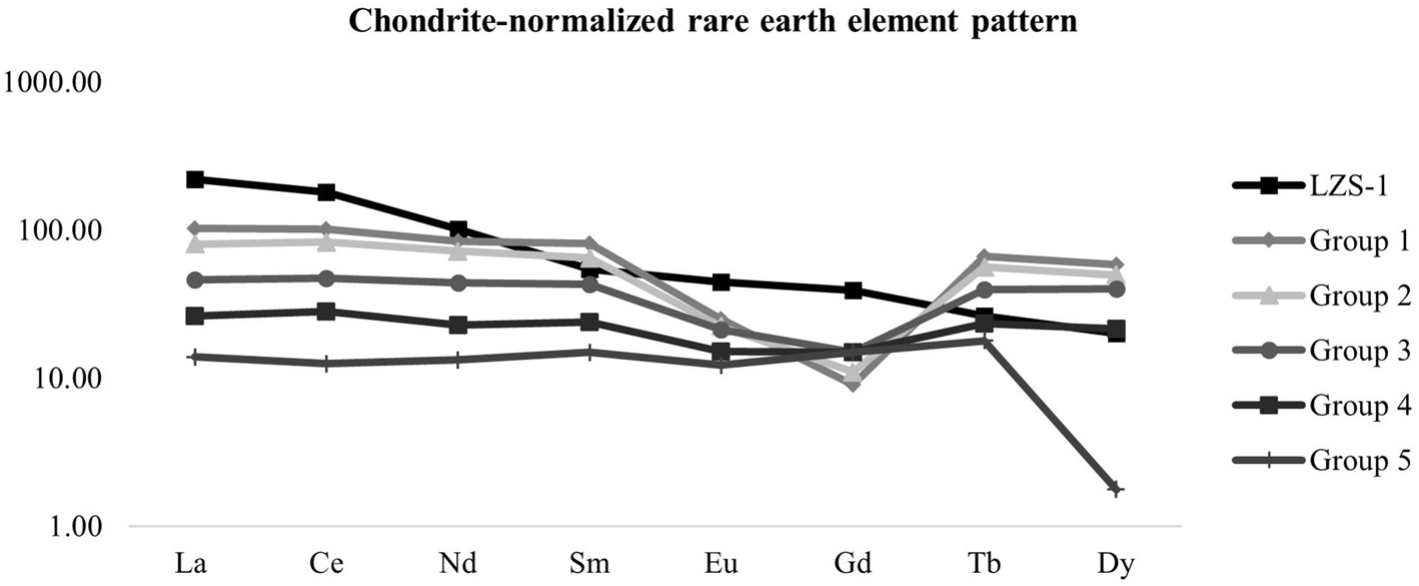
Chondrite-normalized REE distribution pattern of LZS-1 lunar soil simulant and compared with 5 groups of basaltic lunar samples with high Al^25^ normalized to chondrite according to Evensen. et al.^26^.

Trace element composition of LZS-1 was analysed by ICP-MS (Table 3) and found to resemble basanite-basalt. Rare earth element (REE) concentrations are shown in Fig. 8 displaying light REE enrichment though no Ce or Eu anomalies. LZS-1 has high ratios of incompatible elements and is enriched in large ion lithophiles (Fig. 8). This suggests that the material comes from the mantle, without differentiation in the magma chamber.

**Table 3:** Trace element abundances of LZS-1 lunar soil simulant.

Ba 119.83	Sr 1332.43	Zr 529.07	Nb 115.37	Cr 691.37	V 119.07	Sc 36.53	Rb 4.10	Ni 459.07
Co 50.73	Zn 119.03	Cu 114.20	Y 36.37	La 220.77	Ce 180.28	Nd 101.31	Sm 54.55	Eu 44.81
Gd 39.16	Tb 26.17	Dy 20.07	Total 4492.28

Table 4 shows the average composition of main mineral phases of the lunar regolithic simulant LZS-1 compared with five groups of aluminium mare basalt samples from the lunar surface^25^.

**Table 4:** Average composition of major mineral phases in five groups of aluminium mare basalt samples from the lunar surface^25^ and in LZS-1 obtained with the SEM–EDX.

	Average composition of major mineral phases	
	**Group 1**	**LZS-1 (1)**
Plagioclase	An_87.6_Ab_11.2_Or _1.1_	An_73.8_Ab_26.2_Or_0.0_
Pyroxene	En_49.4_Fs_32.5_W_17.4_	En_19.0_Fs_25.0_W_56.0_
Olivine	Fo_65.9_	Fo_68.1_
Metal oxide	Fe_100.0_Ni_0.20_Co_0.45_	Fe_55.64_Ti_3.0_Cr_41.7_
	**Group 2**	**LZS-1 (2)**
Plagioclase	An_87.8_Ab_11.1_Or _1.1_	An_71.8_Ab_24.1_Or_4.1_
Pyroxene	En_48.0_Fs_33.8_W_18.2_	En_19.0_Fs_26.0.8_W_55.0_
Olivine	Fo_65.4_	Fo_69.0_
Metal oxide	Fe_99.6_Ni_0.26_Co_0.75_	Fe_63.6_Ti_5.7_Cr_30.7_
	**Group 3**	**LZS-1 (3)**
Plagioclase	An_90.1_Ab_9.1_Or _0.7_	An_90.7_Ab_6.8_Or_2.5_
Pyroxene	En_49.1_Fs_32.6_W_18.3_	En_22.0_Fs_21.0_W_58.0_
Olivine	Fo_65.5_	Fo_67.2_
Metal oxide	-	Fe_75.7_Ti_17.9_Cr_0.0_
	**Group 4**	**LZS-1 (4)**
Plagioclase	An_90.4_Ab_8.9_Or _0.7_	An_70.7_Ab_24.4_Or_4.9_
Pyroxene	En_48.9_Fs_33.2_W_17.9_	En_22.0_Fs_23.0_W_55.0_
Olivine	Fo_66.4_	Fo_69.1_
Metal oxide	Fe_97.0_Ni_0.50_Co_1.54_	Fe_80.9_Ti_19.1_Cr_0.0_
	**Group 5**	
Plagioclase	An_91.5_Ab_7.7_Or _0.7_	
Pyroxene	En_44.5_Fs_33.2_W_22.2_	
Olivine	Fo_67.9_	
Metal oxide	Fe_99.8_Ni_0.13_Co_0.28_	

There is no evidence of weathering processes in the minerals after eruptive process. No carbonates, quartz or clay have been found that could indicate that the basalts have been altered.

### Physical properties

The physical properties of colour, hardness, ultrasound pulse velocity, density, porosity, and uniaxial compressive strength of LZS-1 have been determined through petrophysical tests. In fact, the values obtained from LZS-1 of density, ultrasound pulse velocity, porosity and uniaxial compressive strength can be compared with a lunar basalt model from Apollo 14^20^ and with the data obtained from the Fra-Mauro formation^27^, which they also correspond to the Apollo 14 mission.

**Figure 9:**
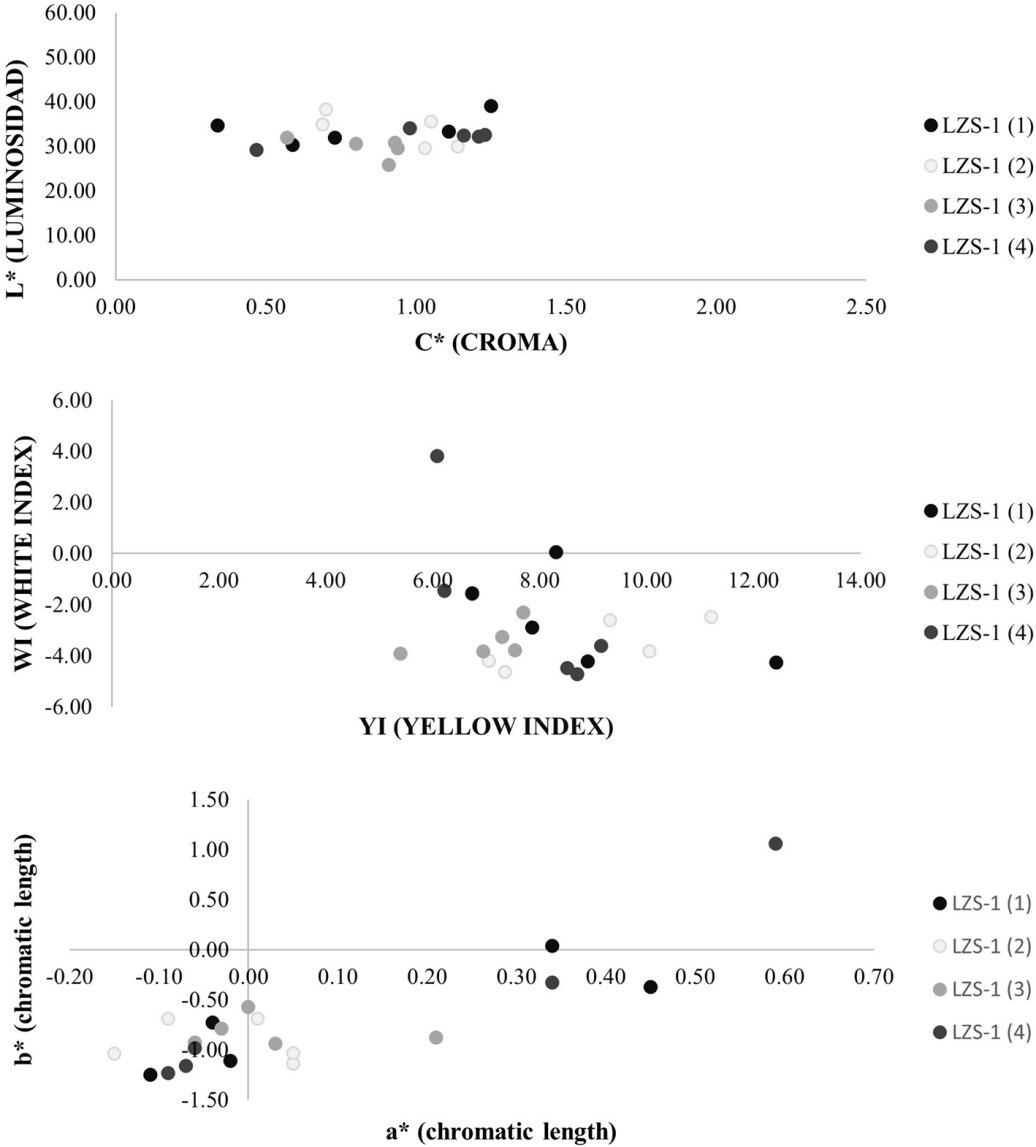
Data from colour test where it can be observed that there is hardly any dispersion between the analysed samples and therefore it can be said that they have not been altered by weathering processes after the eruption.

Colour was measured with a Minolta CM 700d spectrophotometer and COLOR DATA SPECTRAMAGICTM NX CM-S100W software, using Standard D65 illuminant (standard illuminant of the CIE -Commission International de l´Eclaraige-, equivalent to daylight with ultraviolet radiation and a temperature of colour of 6504 ºK) and a viewing angle or observer angle of 10º (Fig. 9).

The CIE L* a* b* system has been used. Where L* is the attribute that determines the degree of luminosity, brightness, or darkness of a colour. Displays values from 0 (pure black) to 100 (pure white). The higher the value, the lighter, and the lower the value, the darker. a * and b * are the chromatic coordinates: the axis -a *, + a * represents the degree of saturation towards green (-a*) and towards red (+ a *). The -b *, + b * axis represents blue to yellow and C* is the Croma obtained with the Eq. (1).

1 $$ C^{*} = \left( {a^{*2 } + b^{*2 } } \right)^{{{\raise0.7ex\hbox{$1$} \!\mathord{\left/ {\vphantom {1 2}}\right.\kern-\nulldelimiterspace} \!\lower0.7ex\hbox{$2$}}}} $$

**Figure 10:**
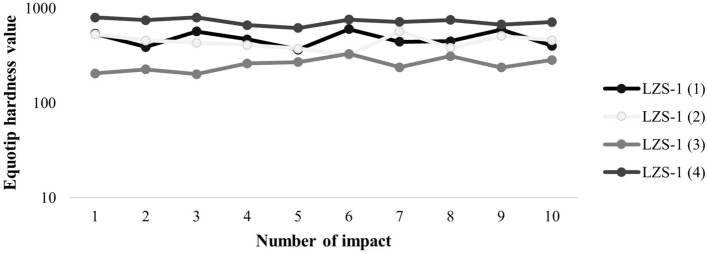
Hardness data from Equotip 3 device.

Hardness (Fig. 10) was measured with the EQUOTIP 3 device according to the LEEB rebound method (EHT, Equotip Hardness Tester).

**Figure 11:**
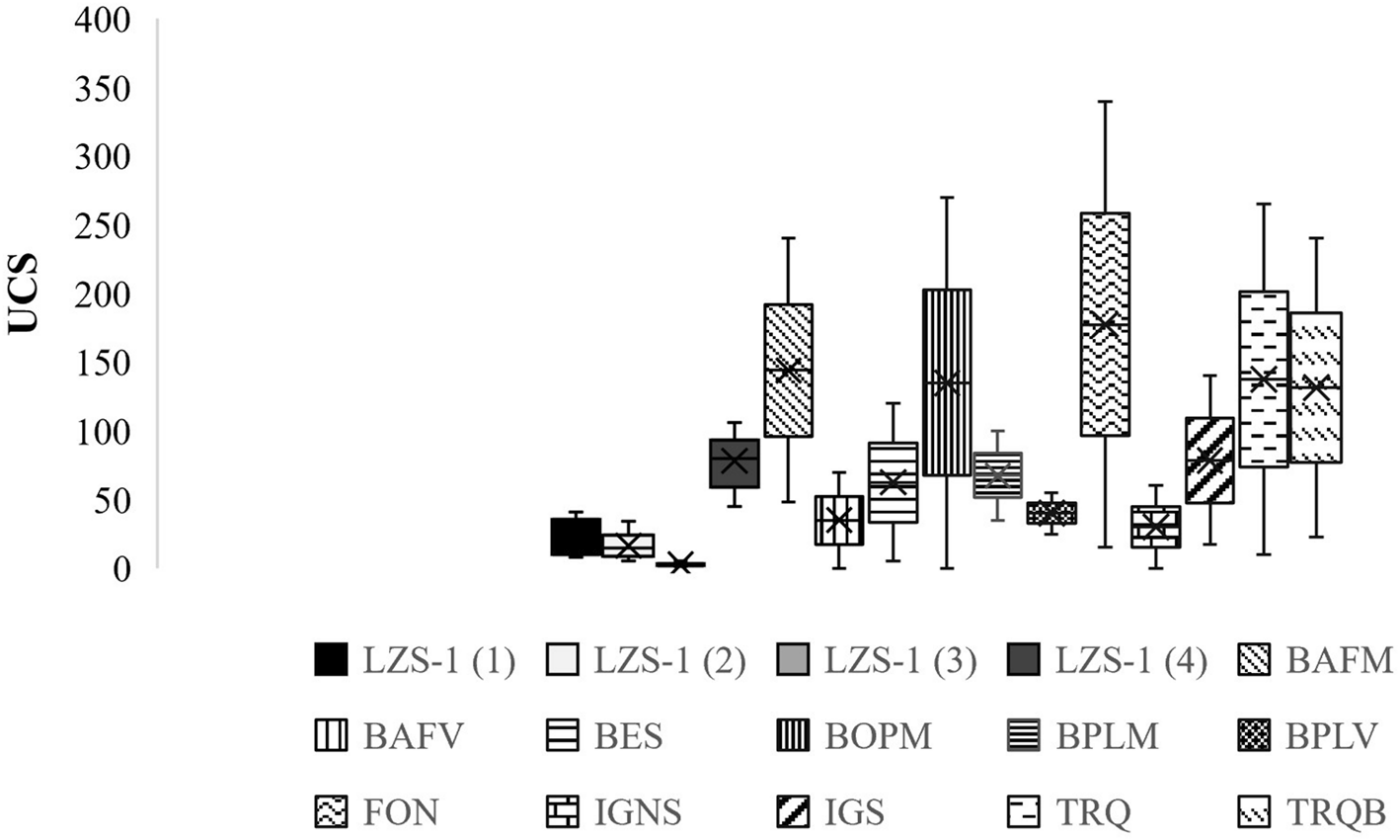
UCS estimation data derived from the equation obtained by Yilmaz. & Goktan^28^. BES Scoriaceous basalt, BAFV Vesicular aphanitic basalt, BOPM Massive pyrogenic olivine basalt, BPLM Massive plagioclassic basalt, FON Phonolite, IGNS Unwelded ignimbrite, TRQ Trachyte, IGS Welded ignimbrite, TRQB Trachybasalt (Modified from^20^).

This measurement is indicative of the surface hardness of the materials used in engineering and has allowed estimations of the uniaxial compressive strength of the samples from the Tao quarry. The results of the uniaxial compressive strength estimation (Fig. 11) were obtained with the Eq. (2).

2 $$ UCS \left( {MPa} \right) = 2*10^{ - 8} *EHT^{3.3492} $$

**Figure 12:**
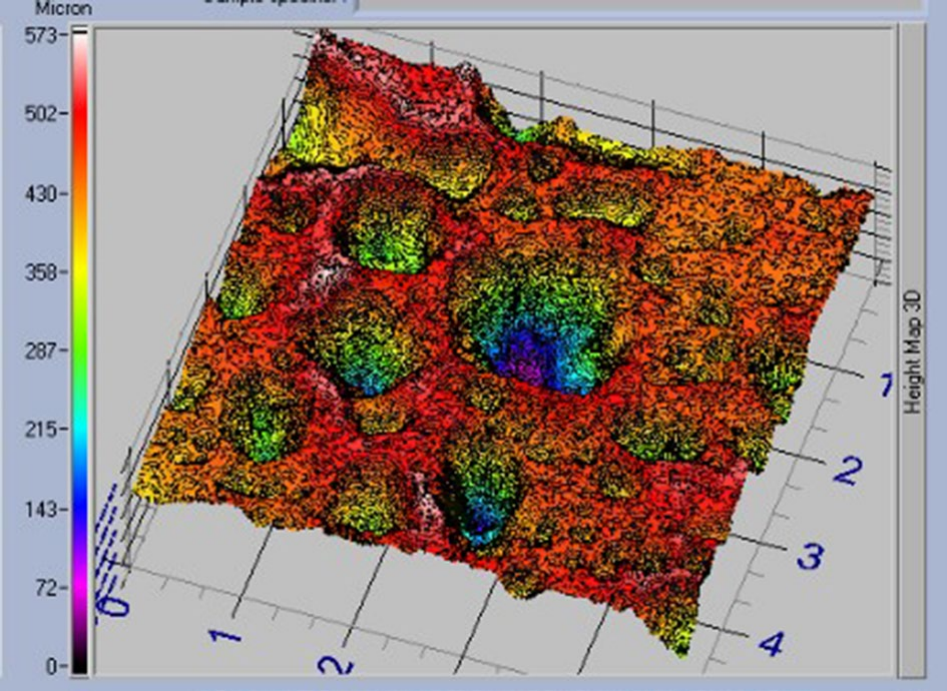
Roughness data from TRACEIT optical surface roughness meter.

The ultrasound pulse velocity represents the global anisotropy (Table 5) of the rock and the roughness the anisotropy at the surface level (Fig. 12).

**Table 5:** Ultrasound pulse velocity test and anisotropy results where Vmed corresponds to: Medium Velocity, StD Med: Medium Standard Deviation, dM: Total Anisotropy and dm: Relative Anisotropy.

Samples	V med	StD med	dM*	dm*
LZS-1_r_ (1)	5271.41	240.69	6.21	1.40
LZS-1_r_ (2)	5594.81	291.49	8.24	4.43
LZS-1_r_ (3)	4740.29	927.52	31.87	13.70
LZS-1_r_ (4)	4817.24	979.35	5.16	2.38

*dM and dm correspond to the total and relative anisotropy of the basalts. r indicates that the test has been performed on a cylindrical rock sample 1–4 correspond to the different measurements on the same sample.

The density and porosity of the rock were calculated through mercury intrusion porosimetry (Micromeritics Autopore IV, with a maximum pressure of 400 MPa). Density values ranged from 2.8 g/cm^3^ to 3.0 g/cm^3^ in all Tao samples. In turn, due to the high penetration of mercury in the rock pores, two porosity values were obtained: microporosity (< 5 µm) and macroporosity (> 5 µm). The results (Table 6) show that the Tao Basalts have a microporosity much higher than the macroporosity.

**Table 6:** Mercury porosimetry test results.

Samples	Mercury global porosity (%)	Real density (g/cm^3^)	Apparent density (g/cm^3^)	Microporosity (%)	Macroporosity (%)
LZS-1_r_ (1)	34.1	3.0	2.0	70.0	30.0
LZS-1_r_ (2)	26.1	2.8	2.0	81.7	18.3

1–2 correspond to the different measurements on the same sample. r indicates that the tests has been performed on a cylindrical rock sample.

The comparison between these results and the data from the Apollo 14 mission^27^ and a lunar basalt model^20^ appear in Table 7. As can be seen, there is a strong correlation not only in terms of mineralogical and geochemical terms, but also in physical properties. For this reason, the Tao basalts are considered analogous to the lunar surface according to the landing site of the Apollo 14 mission.

**Table 7:** Comparison of physical properties between a lunar basaltic model, the Fra Mauro formation, and the LZS-1.

	Apollo 14 lunar basalt model^20^	Fra-Mauro formation^27^	LZS-1
Density (g/cm^3^)	2.9–3.0	2.4–2.5	2.8–3.0
Ultrasound pulse velocity (m/s)	5700	–	5105.9
Porosity (%)	1.8–22.1	17.5–22.0	26.0–34.0
UCS (MPa)	52.0	–	4.1–92.9

## Conclusions

A new basaltic simulant from Lanzarote has been developed, which matches with the Apollo 14 lunar soil. Lanzarote is currently being used as a terrestrial analog for different studies, 29 (ESA-PANGAEA, 2022)^29^. The fabrication of the LZS-1 simulant complements all these activities, opening new research investigations linked to perform additional geological and astrobiological tests (e.g. extraction of oxygen from the basaltic oxides, essays about its potential use as building material and to develop new experiments related to food cultivation on the moon, among others).

## Data Availability

This study was carried out in the framework of a scientific and educational Agreement between the Institute of Geosciences (IGEO) and the *"Cabildo Insular de Lanzarote".* All datasets used and/or analyses carried out and results obtained are available from the corresponding author on reasonable request.

## References

[1] Carrier WD, Olhoeft GR, Mendell W, Heiken GH, Vaniman DT, French BM (1991). Physical properties of thelunar surface. Lunar Source Book.

[2] Lunar, R. Creciendo hacia el espacio próximo a la Tierra. De la mineralogía y recursos terrestres a la exploración planetaria. Discurso pronunciado en el Acto de Toma de Posesión como Académica de Número de la Real Academia de Doctores de España (2006).

[3] Anand M, Crawford IA, Balat-Pichelin M, Abanades S, Van Westrenen W, Péraudeau G, Seboldt W (2012). A brief review of chemical and mineralogical resources on the Moon and likely initial in situ resource utilization (ISRU) applications. Planet. Space Sci..

[4] Kanamori, H., Udagawa, S., Yoshida, T., Matsumoto, S., & Takagi, K. Properties of lunar soil simulant manufactured in Japan. In *Proceeding of the Sixth International Conference and Exposition on Engineering Construction, and Operations in Space. American Society of Civil Engineers, Albuquerque* (1998).

[5] Zheng Y, Wang S, Ouyang Z, Zou Y, Liu J, Li C, Feng J (2009). CAS-1 lunar soil simulant. Adv. Space Res..

[6] Weiblen, P.W., & Gordon, K. Characteristics of a simulant for lunar surface materials. In *Proceeding of the Second Conference on Lunar Bases and Space Activities of the 21st Century. Lunar and Planetary Institute, Houston* (1988).

[7] Alberquilla F (2021). Peñas de Tao geosite: an open door to the recent volcanic history of Lanzarote and a step towards the Moon. Int. J. Earth Sci. (Geol Rundsch).

[8] Romero C (2003). El relieve de Lanzarote.

[9] Romero, C., Sánchez, N., Vegas, J., & Galindo, I. Historic volcanic landforms diversity on lanzarote. In *Lanzarote and Chinijo Islands Geopark: From Earth to Space *47–73 (Springer, Cham, 2019).

[10] Martínez JE, Mariñoso PE (2020). Timanfaya lava flows geosite: a historical and educational approach. Int. J. Earth Sci..

[11] Carracedo JC, Rodríguez Badiola E (1993). Evolución geológica y magmática de la isla de Lanzarote (Islas Canarias). Revista de la Academia Canaria de la Ciencia.

[12] Shearer CK, Papike JJ (2005). Early crustal building processes on the moon: Models for the petrogenesis of the magnesian suite. Geochim. Cosmochim. Acta.

[13] Martínez Frías J, Mateo Mederos ME, Lunar Hernández R (2016). Los geoparques como áreas de investigación, geoeducación y geoética en geociencias planetarias: el geoparque de Lanzarote y Archipiélago Chinijo. Geotemas.

[14] Davies, F. T., He, C., Lacey, R. E., & Ngo, Q. Growing plants for NASA–challenges in lunar and Martian agriculture. In *Combined Proceedings International Plant Propagators’ Society *Vol. 53, pp. 59–64 (2003)

[15] Schwandt C, Hamilton JA, Fray DJ, Crawford IA (2012). The production of oxygen and metal from lunar regolith. Planet. Space Sci..

[16] Neal CR, Taylor LA (1992). Petrogenesis of mare basalts: A record of lunar volcanism. Geochim. Cosmochim. Acta.

[17] Martínez-Frías J, Mateo-Mederos ME, Lunar Hernández R (2017). The scientific and educational significance of geoparks as planetary analogues: the example of Lanzarote and Chinijo Islands UNESCO Global Geopark. Episodes.

[18] McKay, D.S., Carter, J.L., Boles, W.W., Allen, C.C., & Allton, J.H. JSC-1: A new lunar regolith simulant. In *Proceeding of the 24th Lunar and Planetary Science Conference. Lunar and Planetary Institute, Houston* 963–964 (1993).

[19] Li, Y., Liu, J., & Yue, Z. NAO-1: Lunar highland soil simulant developed in China. *Aerospace Engineering*, **22**(1). (2009)

[20] Rodríguez-Losada, J. A., Hernández-Fernández, S., Martínez-Frías, J., Hernández, L. E., & Lunar Hernández, R. Study of lunar soil from terrestrial models (Canary Islands, Spain). In *ISRM International Workshop on Rock Mechanics and Geoengineering in Volcanic Environments. International Society for Rock Mechanics and Rock Engineering*, vol. 6 (2010).

[21] Pantazidis A, Baziotis I, Solomonidou A, Manoutsoglou E, Palles D, Kamitsos E, Asimow PD (2019). Santorini volcano as a potential Martian analogue: The Balos Cove Basalts. Icarus.

[22] Katagiri J, Matsushima T, Yamada Y, Tsuchiyama A, Nakano T, Uesugi K, Saiki K (2015). Investigation of 3D grain shape characteristics of lunar soil retrieved in Apollo 16 using image-based discrete-element modeling. J. Aerosp. Eng..

[23] Heiken GH, Vaniman DT, French BM (1991). Lunar Sourcebook – AUser’s Guide to the Moon.

[24] McKay, D.S., Carter, J.L., Boles, W.W., Allen, C.C., & Allton, J.H. JSC-1: anew lunar soil simulant, In *Proceeding of the Fourth Engineering, Construction, and Operations in Space, American Society of Civil Engineers, Albuquerque*, 857–866 (1994).

[25] Dickinson T, Taylor GJ, Keil K, Schmitt RA, Hughes SS, Smith MR (1985). Apollo 14 aluminous mare basalts and their possible relationship to KREEP. J. Geophys. Res. Solid Earth.

[26] Evensen NM, Hamilton PJ, O'nions, R. K. (1978). Rare-earth abundances in chondritic meteorites. Geochim. Cosmochim. Acta.

[27] Kiefer WS, Macke RJ, Britt DT, Irving AJ, Consolmagno GJ (2012). The density and porosity of lunar rocks. Geophys. Res. Lett..

[28] Yilmaz NG, Goktan RM (2019). Comparison and combination of two NDT methods with implications for compressive strength evaluation of selected masonry and building stones. Bull. Eng. Geol. Env..

[29] ESA-PANGAEA. https://www.esa.int/Science_Exploration/Human_and_Robotic_Exploration/CAVES_and_Pangaea/What_is_Pangaea (2022)

